# Enhancing the stature of JIAPS: Our immediate plans

**DOI:** 10.4103/0971-9261.42563

**Published:** 2008

**Authors:** K. L. Narasimhan

**Affiliations:** Editorial Secretary, JIAPS, PGIMER, Chandigarh, India. E-mail: narasimhankl@gmail.com

I wish to thank Prof. K. L. N. Rao, Professor & Head, Dept. of Pediatric Surgery, my teacher and mentor, for asking me to be the Editorial Secretary of JIAPS during his tenure as The Editor-in-Chief. The Department of Pediatric Surgery at PGI, Chandigarh has been fortunate to be lead earlier by eminent men like Prof. I. C. Pathak, Prof. S. K. Mitra, and now by Prof. Rao. We have been lucky that we have a reasonable infrastructure and a large number of trainees and a huge population of sick children (around 150 esophageal atresias operated per year and that too, almost all of it by trainees under supervision). Even with all these, we find it difficult to do basic research and we are just able to do some Clinical Research. Hence, the majority of the pediatric surgeons in this country who are outside the Institutions (90% of the pediatric surgeons) cannot find the resource, clinical material, or the time to do research or publish papers. Yet, we are encouraged to see that a large number of Community Pediatric Surgeons of India are trying to communicate to the rest of the world through JIAPS. This gives us hope that our journal will get indexed in the near future.

About the future of the journal, we have two immediate objectives in our mind: (1) To get the journal indexed, (ii) To improve the finances of our journal. The indexing of the journal is possible only if outside peer reviewers in our field think that our journal is of a particular scientific standard laid down. Hence, we need series publications of good quality to be sent to the journal instead of case reports. It is for the good of all members of IAPS that we are requesting this favor. The editorial board is also thinking of financially rewarding the best papers every year. All the M.Ch. trainees are requested to submit their research work as a paper to the JIAPS.

Prof. Rao has thought of an innovative idea to increase the number of good articles in the journal. All papers presented in the regional/zonal meeting of IAPS may be sent for consideration for publishing in the journal. These will be published without delay. We will help actively in improving the quality of the content of the articles by corresponding with the authors. Our idea is to make JIAPS a quality journal of the developing world. We also would like to bring out articles for the trainees – a trainee's corner, meant to be oriented toward the trainees and their exams. A practitioner's column, where interesting anecdotes or interesting cases are discussed, is also planned. This column is open only for practitioners in pediatric surgery not affiliated to teaching institutions.

We would also like to see JIAPS as a forum, where the pediatric surgeons of the developed world, which is lacking the clinical material (due to low birth rate, and decreasing fetal anomalies because of antenatal intervention), interact with the developing world. The pediatric surgeons of the developing world are innovative and combat the resource crunch effectively to keep the community of sick children happy. Interaction between the surgeons of the two worlds will be mutually beneficial, as the trainee in the developed world does not get to see enough clinical material, whereas the trainee in the developing world does not get to see sophisticated infrastructure. We will have an interactive case presentation column, where we will have to pose difficult cases to eminent surgeons from renound centers of pediatric surgery. We would like to see the journal achieve the objectives during the current tenure when the journal office is in Chandigarh, and we solicit your cooperation and help. We would like to receive letters to editors on specific topics like (1) Introduction to health insurance for children, (2) Sub-specialization in Pediatric Surgery, and (3) Content of training/quality of training received in Pediatric Surgery by trainees. We would also like to have feedbacks regarding the journal e-mailed to us periodically. We request you, all the members of IAPS, to work to enhance the stature of our journal.

